# Dietary Antioxidant Trans-Cinnamaldehyde Reduced Visfatin-Induced Breast Cancer Progression: In Vivo and In Vitro Study

**DOI:** 10.3390/antiox8120625

**Published:** 2019-12-06

**Authors:** Yi-Fen Chiang, Hsin-Yuan Chen, Ko-Chieh Huang, Po-Han Lin, Shih-Min Hsia

**Affiliations:** 1 Graduate Institute of Metabolism and Obesity Sciences, College of Nutrition, Taipei Medical University, Taipei 11031, Taiwan; yvonne840828@gmail.com; 2 School of Nutrition and Health Sciences, College of Nutrition, Taipei Medical University, Taipei 11031, Taiwan; hsin246@gmail.com (H.-Y.C.); a910241@gmail.com (K.-C.H.); phlin@tmu.edu.tw (P.-H.L.); 3 School of Food and Safety, College of Nutrition, Taipei Medical University, Taipei 11031, Taiwan; 4 Nutrition Research Center, Taipei Medical University Hospital, Taipei 11031, Taiwan

**Keywords:** visfatin, cinnamaldehyde, breast cancer, adipokine

## Abstract

Excessive growth of cancer cells is the main cause of cancer mortality. Therefore, discovering how to inhibit cancer growth is an important research topic. Recently, the newly discovered adipokine, known as nicotinamide phosphoribosyl transferase (NAMPT, visfatin), which has been associated with metabolic syndrome and obesity, has also been found to be a major cause of cancer proliferation. Therefore, inhibition of NAMPT and reduction of Nicotinamide adenine dinucleotide (NAD) synthesis is one strategy for cancer therapy. Cinnamaldehyde (CA), as an antioxidant and anticancer natural compound, may have the ability to inhibit visfatin. The breast cancer cell line and xenograft animal models were treated under different dosages of visfatin combined with CA and FK866 (a visfatin inhibitor) to test for cell toxicity, as well as inhibition of tumor-related proliferation of protein expression. In the breast cancer cell and the xenograft animal model, visfatin significantly increased proliferation-related protein expression, but combination with CA or FK866 significantly reduced visfatin-induced carcinogenic effects. For the first time, a natural compound inhibiting extracellular and intracellular NAMPT has been demonstrated. We hope that, in the future, this can be used as a potential anticancer compound and provide further directions for research.

## 1. Introduction

Breast cancer is the most common gynecological cancer, impacting over 2.1 million women per year, according to the World Health Organization (WHO) [1]. In 2018, breast cancer caused 15% of all cancer deaths among women (WHO, 2018). Therefore, preventing breast cancer has become an important issue. Environmental, genetic, and other factors may increase the risk of breast cancer. In addition, female hormones linked to increasing age are associated with promoting cancer cell proliferation and increasing cancer risk. Yet, the increase in adipose tissue caused by obesity is the most important risk factor [2]. Obesity is a multifactorial disease that corresponds with the environment, behavior, and heredity. Previous studies have shown a positive correlation between Body Mass Index (BMI) (for each additional 5 kg/m^2^) and breast cancer risk [3]. Obesity is also accompanied by changes in adipokines [4]. 

In cancer research, visfatin has been shown to be a potential biomarker, with higher serum visfatin levels in cancer patients than in a healthy control group [5,6]. Visfatin, which is also known as NAMPT or pre–B-cell colony-enhancing factor (PBEF), is a 52 kDa adipocytokine that has both intracellular (iNAMPT) and extracellular (eNAMPT) forms [7]. eNAMPT has proliferative, inflammatory, and anti-apoptotic effects via increased reactive oxygen species (ROS). Visfatin can increase cell adhesion ability [8,9,10,11], and intracellular cancer cell visfatin has been shown to increase cell proliferation through Protein kinase B(Akt) /phosphoinositide 3-kinases (PI3K) and extracellular-signal-regulated kinase/ mitogen activated protein kinase (ERK/MAPK) activation [12]. For cancer cell proliferation, energy metabolism plays an important role: NAD^+^ is important for energy-metabolic pathways and plays a role in cancer cell progression [13]. Nicotinamide phosphoribosyl transferase (NAMPT) is a rate-limiting enzyme for the NAD^+^ salvage pathway [14]. Thus, it is important to discover a compound that inhibits the effects of visfatin. Recently, research has focused on means of inhibiting visfatin to reduce cancer progression. However, few studies have been conducted on the ability of natural compounds to inhibit visfatin. 

Trans-cinnamaldehyde (CA) is an active ingredient and antioxidant derived from cinnamon, which has, for a long time, been used as a natural spice in cooking and in the industry [15,16]. CA is the source of the characteristic aroma of cinnamon, and it is found in concentrations of 1% to 4% in cinnamon bark. Of the many compounds in cinnamon oil, the most abundant is CA, accounting for 65% to 75% of all compounds. It is mainly used as a flavoring agent or for providing the aroma of candles. It has antiviral, antibacterial, and antifungal properties [17].

CA has shown anticancer effects on lung cancer, rectal cancer, and oral cancer in vitro and in vivo [18,19,20,21]. It has a similar structure to curcumin, which influences the inhibition of visfatin protein expression [22].

The aim of this research is to enhance our understanding of the ability of CA to reduce visfatin protein expression and activity, and its effects on visfatin-induced cancer progression. We demonstrate the effect of CA on inhibiting iNAMPT, eNAMPT, and related pathways, which shows its anticancer effects.

## 2. Materials and Methods

### 2.1. Reagents

Trans-cinnamaldehyde was obtained from Sigma-Aldrich (St. Louis, MO, USA). FK866 (visfatin inhibitor) was obtained from Cayman chemical company (Ann Arbor, MI, USA). Human recombinant visfatin was obtain from Peprotech (#130-09, Rehovot, Israel).

### 2.2. Systemic Review

We first undertook a systemic review, using keywords containing (“Nicotinamide Phosphoribosyl transferase” [Mesh] AND “cancer” [Mesh] OR “Breast Neoplasms” [Mesh]). We used PubMed as the search engine of study titles. Then the 351 articles chosen matching keywords were screened by abstract reading, excluding in vitro studies, non-clinical studies, and analyses without enzyme-linked immunosorbent assay (ELISA) kits.

### 2.3. Meta-Analysis

Lastly, six studies were screened for analysis [5,23,24,25,26,27], which included a meta-analysis and the use of R studio software. These were analyses of serum visfatin concentrations in breast cancer patients and healthy subjects from different ethnicities and regions.

### 2.4. Survival Rate

To understand whether visfatin influences the clinical physiology of breast cancer patients in terms of survival, the Kaplan–Meier estimator was used. The database in Reference [28] was used to analyze the survival rate of the visfatin gene (217738_at) in breast cancer patients, where a total of 869 patients with ER-negative breast cancer were screened.

### 2.5. Cell Culture

The human breast cancer cell line MDA-MB-231-2A- Green fluorescent protein (GFP) (SC044, MDA-MB-231-GFP) was obtained from GenTarget (San Diego, CA, USA) and a normal epithelial cell line 184B5 was obtained from the Bioresourse Collection and Research Center (BCRC, Hsinchu, Taiwan). Cell lines were cultured in Dulbecco’s Modified Eagle Medium: Nutrient Mixture F-12 (DMEM F12) (CAISSON, Taichung City, Taiwan) or DMEM (Thermo Fisher Scientific, Waltham, MA, USA) supplemented with 10% fetal bovine serum (FBS) (Gibco, Grand Island, NY, USA) and 100X penicillin streptomycin solution (CORNING, Manassas, VA, USA) at 37 °C in a humidified 5% CO_2_ incubator.

For treatments, cells were cultured in 96-well plates (3000 cells/ well) or in a 10-cm dish (7 × 10^5^ cells), and then starved with 1% FBS medium for 24 h and incubated with different treatments for 72 h.

### 2.6. 3-(4,5-dimethylthiazol-2-yl)-2,5-diphenyl tetrazolium bromide (MTT) Cell Viability Assay

The 3-(4,5-dimethyl thiazol)-2,5-diphenyltetrazolium bromide (MTT) (Abcam, MA, USA) was decomposed into mitochondrial succinate dehydrogenase in living cells to produce a blue-violet crystal product [29]. After dissolving the crystals in dimethyl sulfoxide (DMSO) (Echo Chemical Co. Ltd., Miaoli, Taiwan) solution, cell survival was judged based on absorbance. A quantity of 3 × 10^3^ cells was suspended in 100 μL of the culture medium and seeded into a 96-well cell culture dish. The next day, cells were starved for 24 h, and cinnamaldehyde (CA) diluted in 1% FBS was added to the culture. After treatments, 100 μL of the 0.1 mg/mL MTT was added, and the mixture was kept in a 37 °C CO_2_ incubator for 4 h, after which 100 μL of dimethyl sulfoxide (DMSO) was added. The effect was to dissolve the blue-violet crystal. An ELISA reader (Molecular Devices, San Jose, CA, USA) was then used to set the optical density (OD). Wavelengths of 570 and 630 nm were used to detect the absorbance.

### 2.7. Cell Counting

MDA-MB-231-GFP cells were seeded in a 6-well plate (2 × 10^5^ cells/well). The next day, cells were starved for 24 h. After the treatment, cells were detected by trypsinization and resuspended with the culture medium. Cells were then stained with trypan blue (CORNING, Christiansburg, VA, USA). The cells were counted with a hemocytometer under a microscope (Olympus, Tokyo, Japan).

### 2.8. Colony Formation

MDA-MB-231-GFP cells were seeded in a 6-well plate (500 cells/well), and treated with CA or FK866, or combined with visfatin for 72 h. Then, cells were removed from the medium, washed with PBS twice, placed in completed medium, and cultured for one week. Colonies were fixed with 95% methanol (Echo Chemical Co. Ltd., Miaoli, Taiwan) and stained with 0.5% crystal violet [30].

### 2.9. Western Blot Analysis

MDA-MB-231-GFP cell lysates were prepared in ice-cold lysis buffer (50 mmol/L Tris (pH 8.0), 100 mmol/L sodium chloride (NaCl), 0.1% sodium dodecyl sulfate (SDS), 1% NP-40, and 0.5 mM ethylene diamine tetra acetic acid (EDTA) containing the protease (Roche, Basel, Switzerland), the phosphatase inhibitor (Roche, Basel, Switzerland) cocktail, or both. The proteins (25 μg) were boiled for 5 min, separated using 7.5% or 15% SDS-polyacrylamide gel electrophoresis (PAGE), and then transferred electrophoretically onto Immobilon-P polyvinylidene fluoride (PVDF) membranes (0.22 µm) for 150–180 min at 280 mA and 250 V. Then, the membranes were washed three times for 10 min each with Tris-buffered saline (TBS) plus Tween 20 (TBST) buffer, blocked with blocking buffer (5% BSA) for 1 h at room temperature, and incubated overnight with primary antibodies (Table S1) Nicotinamide phosphoribosyl transferase (NAMPT) (Proteintech, Rehovot, Israel), proliferating cell nuclear antigen (PCNA) (Cell signaling, Danvers, MA, USA), phospho-mammalian target of rapamycin (p-mTOR) (Cell signaling, Danvers, MA, USA), mTOR (Cell signaling, Danvers, MA, USA), phosphor-phosphoinositide 3-kinase (p-PI3K) (Cell signaling, Danvers, MA, USA), PI3K (Cell signaling, Danvers, MA, USA), and glyceraldehyde-3-phosphate dehydrogenase (GAPDH) (Proteintech, Rehovot, Israel) at 4 °C. The next day, the membranes were washed three times for 10 min each with TBST (Tris Buffered Saline with Tween 20) buffer, incubated for 1 h in blocking buffer with anti-rabbit/mouse IgG coupled to alkaline phosphatase (1:10,000), and washed three times (10 min each time) with TBST buffer. Then, the bands were detected using enhanced chemiluminescence (ECL). The values shown were quantified, normalized to the internal control GAPDH, and then densitometry estimation was performed using ImageJ software (NIH, Bethesda, MD, USA) [31].

### 2.10. Nicotinamide phosphoribosyl transferase (NAMPT) Activity Kit

Analysis of enzyme activity was conducted using a commercial analysis kit (Abcam, ab221819). A mixture of ATP, nicotinamide mononucleotide adenylyltransferase (NMNAT), nicotinamide, phosphoribosyl pyrophosphate (PRPP), and the compound was incubated for 30 min. WST-1, ADH, diaphorase, and ethanol solution was added. Output was measured at OD 450 nm on a microplate reader in a kinetic mode, every 5 min, for at least 30 min, at 30 °C, which is protected from light.

### 2.11. Xenograft Animal Model

Tumor xenograft was undertaken in nude mice. Five-week-old female Balb/c nude mice (BioLASCO, Taipei, Taiwan) were housed under a 12 h light/12 h dark cycle in a pathogen-free environment, with food and water available ad libitum. Tumors were implanted by subcutaneous injection of MDA-MB-231-GFP cells (2 × 10^6^ suspended in 0.1 mL PBS for each mouse) into the flank of mice. After 1 week, mice were randomly divided into four groups (*n* = 3) and received intraperitoneal injections of visfatin (2 ng/g), CA (100 mg/kg), or FK866 (4 mg/kg) [32] for 56 days. Tumor volume was measured with calipers. Tumor detection was carried out by intraperitoneal injection with 150 mg/kg luciferin, and the tumor was detected using an in vivo imaging system (IVIS). All animal studies were conducted according to the protocols approved by the Institutional Animal Care and Use Committee (IACUC) of Taipei Medical University (IACUC Approval No. 2019-0034).

### 2.12. Immunohistochemistry Analysis

Tumor tissues were embedded, sliced, and stained by Bio-Check Laboratories Ltd. (Taipei, Taiwan). Lastly, a concentration of proliferating cell nuclear antigen (PCNA) (Cell signaling, Danvers, MA, USA) was incubated at a ratio of 1:2000. To analyze the immunohistochemistry slides, they were photographed at 40× magnification using an EVOS^®^ microscope (Thermo Fisher Scientific, Waltham, MA, USA), and a Fiji ImageJ IHC toolbox was used to analyze the colored area of PCNA.

### 2.13. Statistical Analysis

The experimental data are expressed as mean ± standard deviation (SD) and mean ± standard error of the mean (SEM). Statistical analysis was performed using GraphPad Prism version 6 (GraphPad Software, Inc., San Diego, CA, USA). Student’s t-test and one-way analysis of variance (ANOVA) were analyzed and compared using Tukey’s test for post-mortem analysis. The results were considered statistically significant at *p* < 0.05.

## 3. Results

### 3.1. Meta-Analysis of Breast Cancer Patient Visfatin Concentrations

**Figure 1:**
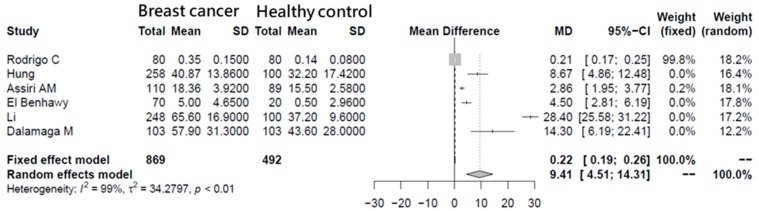
Meta-analysis of breast cancer visfatin concentrations. Forest plot showing the serum visfatin levels between breast cancer and healthy groups. MD: mean difference.

A meta-analysis was carried out in which visfatin concentrations were compared between breast cancer patients (*n* = 869) and a healthy control (*n* = 492). After the included six original articles, because of the variation between different articles (*I^2^* = 99%; *p* < 0.01), a random effects model was applied. The result shows that, when the random effects model was used, the mean difference (MD) of visfatin plasma concentrations was significantly higher in breast cancer patients than in healthy subjects (MD = 9.41, 95% confidence interval (CI) = 4.51–14.31), which indicates the importance of visfatin in breast cancer patients (Figure 1).

### 3.2. Breast Cancer Patient Visfatin Gene Expression and Survival Rate

**Figure 2:**
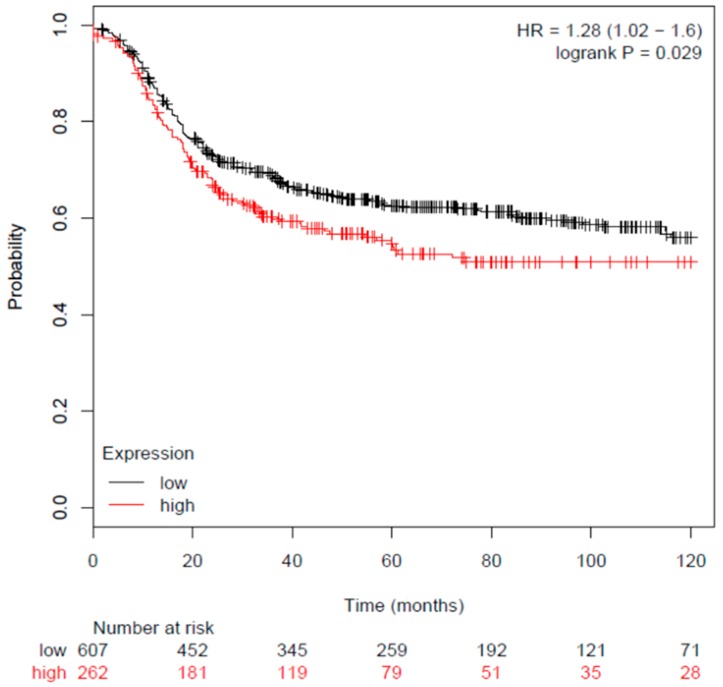
Breast cancer survival and visfatin gene expression. KMplot was used to analyze visfatin gene expression (217738_at) in breast cancer patients, where a total of 869 patients with ER-negative breast cancer were screened (*n* = 869).

To understand whether the visfatin gene expression of breast cancer patients and its correlation with the survival rate, the latter was estimated by a Kaplan–Meier estimator. The study database in Reference [28] was used to analyze the survival rate in breast cancer patients who expressed low/high visfatin genes (217738_at) in which 869 patients with estrogen receptor (ER)-negative breast cancer were included. According to the database analysis, patients with a higher expression (*n* = 262) of the visfatin gene expression compared with lower expression of visfatin gene expression (*n* = 607) had significantly lower survival rates (hazard ratio (HR) = 1.28 (1.02–1.6), *p* = 0.029) (Figure 2).

### 3.3. Effects of cinnamaldehyde (CA) on Visfatin-Induced Breast Cancer Cell

#### 3.3.1. Effect of Visfatin on Breast Cancer Cell Viability

**Figure 3:**
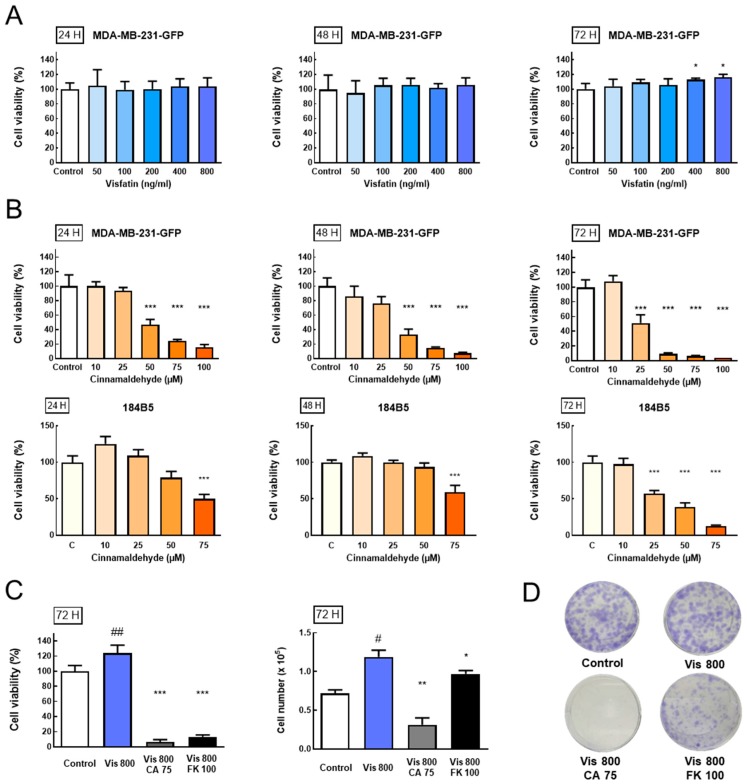
Effects of cinnamaldehyde (CA) and visfatin on the growth of the breast cancer cell line MDA-MB-231-GFP. (**A**) Cell viability. MDA-MB-231-GFP cells were treated with various concentrations of visfatin (*n* = 3). (**B**) Cell viability. MDA-MB-231-GFP and 184B5 cells were treated with various concentrations of CA (*n* = 3). *** *p* < 0.001 compared with the control group. (**C**) Cell viability and cell counting. MDA-MB-231-GFP were treated with CA, FK866 combined with visfatin for 72 h (*n* = 3), and (**D**) colony formation. Effects of CA, FK866 combined with visfatin on MDA-MB-231-GFP cells after 72 h and maintained for one week. # *p* < 0.05, ## *p* < 0.01 compared with the control group. * *p* < 0.05, ** *p* < 0.01, *** *p* < 0.001 compared with the Vis 800 group.

To explore the visfatin effect on cell viability, the MTT assay was used to investigate the cell viability. MDA-MB-231-GFP human breast cancer cells were treated with different concentrations of visfatin (0, 50, 100, 200, 300, 400, and 800 ng/mL). The result shows that visfatin 800 ng/mL significantly increased cell viability after 72 h (Figure 3A) (*p* < 0.05).

#### 3.3.2. Effect of CA on Breast Cancer Cell Viability

To investigate whether CA has an inhibitory effect on the cell viability of MDA-MB-231-GFP, human breast cancer cells and normal epithelial cells 184B5 were treated with different concentrations of CA (0, 10, 25, 50, 75, and 100 μM) and tested by the MTT assay after 24, 48, and 72 h to analyze cell viability. The results showed that administration of 25–100 μM CA significantly inhibited MDA-MB-231-GFP cell viability compared with the control group (Figure 3B, Figure S1A) (*p* < 0.01). To evaluate CA’s effect on normal breast epithelial cells, the normal epithelial cells 184B5 was treated with different concentrations of CA (0, 10, 25, 50, and 75 μM). The result showed that a high dose of CA may cause cell death. However, at such a high dose, CA caused more serious cell death in MDA-MB-231-GFP human breast cancer cells (72 h, 75 μM, 184B5 group: 12.8% ± 1.10% vs. MDA-MB-231-GFP: 4.33% ± 0.57%, *p* < 0.05) (Figure 3B, Figure S2).

#### 3.3.3. Anti-Proliferative Effect of cinnamaldehyde (CA) and FK866 Combined with Visfatin-Induced Breast Cancer Cell Viability

After screening the concentrations of visfatin and CA, the effects of combinations on cell viability of MDA-MB-231-GFP cells were further explored. In addition, trypan blue was used for cell counting. After 72 h of treatment, the results showed that the visfatin group significantly increased cell viability when compared with the control group (visfatin group: 124% ± 10.44% vs. control group: 100% ± 7.54%, *p* < 0.05). In addition, the cell number of the visfatin group also increased significantly when compared with the control group (visfatin group: 117,000 ± 6000 cells vs. control group: 72,000 ± 3000 cells, *p* < 0.01). However, both CA and FK866 significantly decreased visfatin-induced cell viability when compared with the visfatin group (CA group: 6.33% ± 3.05% versus visfatin group: 124% ± 10.44%, *p* < 0.05), (FK group: 13.33% ± 2.30% vs. visfatin group: 124% ± 10.44%, *p* < 0.05), and cell number (CA group: 32,000 ± 6000 cells vs. visfatin group: 117,000 ± 6000 cells, *p* < 0.05), (FK group: 97,000 ± 3000 cells vs. visfatin group: 117,000 ± 6000 cells, *p* < 0.05) (Figure 3C, Figure S3).

#### 3.3.4. Anti-Colony Effect of CA Combined with Visfatin-Induced Breast Cancer Cells

Colony formation is a cancer proliferation marker. After 72 h of treatment and culturing for 1 week, cell aggregation ability was analyzed by colony formation to understand the inhibitory effects of CA on visfatin-induced cell proliferation. The results showed that, after treatment with visfatin 800 ng/mL for 72 h following 1 week of cell culturing, the colony size was increased, as compared with the control group. The combined intervention of CA and FK866 significantly inhibited visfatin-induced cell colony formation (Figure 3D).

### 3.4. Effects of CA and FK866 Combined with Visfatin on Proliferation-Related Protein Expression

**Figure 4:**
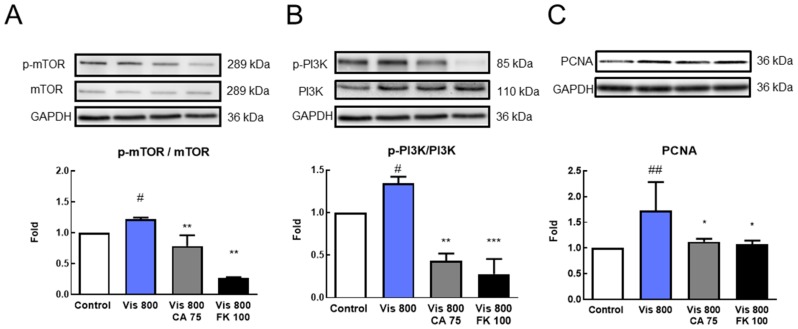
Cinnamaldehyde (CA) and FK866 inhibiting visfatin-induced proliferation-related protein expression. MDA-MB-231-GFP cells were starved for 24 h and then treated with visfatin, CA, and FK866 for 72 h (*n* = 3). Western blot was used to examine the protein expression (**A**) phospho-mammalian target of rapamycin (p-mTOR) (**B**) phosphor-phosphoinositide 3-kinase (p-PI3K) (**C**) proliferating cell nuclear antigen (PCNA). # *p* < 0.05, ## *p* < 0.01 compared with the control group. * *p* < 0.05, ** *p* < 0.01, *** *p* < 0.005 compared with Vis 800 group.

The results of the study on the combination of visfatin, CA, and FK866 found that the administration of visfatin alone significantly increased cell numbers and cell viability, while combination with CA and FK866 had an anti-tumor effect. Thus, we analyzed the related protein expressions. After treatment for 72 h, the results showed that visfatin significantly increased the expression of proliferative-associated proteins p-mTOR, mTOR, p-PI3K, PI3K, and PCNA when compared with the control group (*p* < 0.05). The combination of CA and FK866 significantly reduced the proliferative-associated proteins induced by visfatin (*p* < 0.05) (Figure 4).

### 3.5. Effects of CA and FK866 Combined with Visfatin on Intracellular and Extracellular Nicotinamide phosphoribosyl transferase (NAMPT) Protein Expression

**Figure 5:**
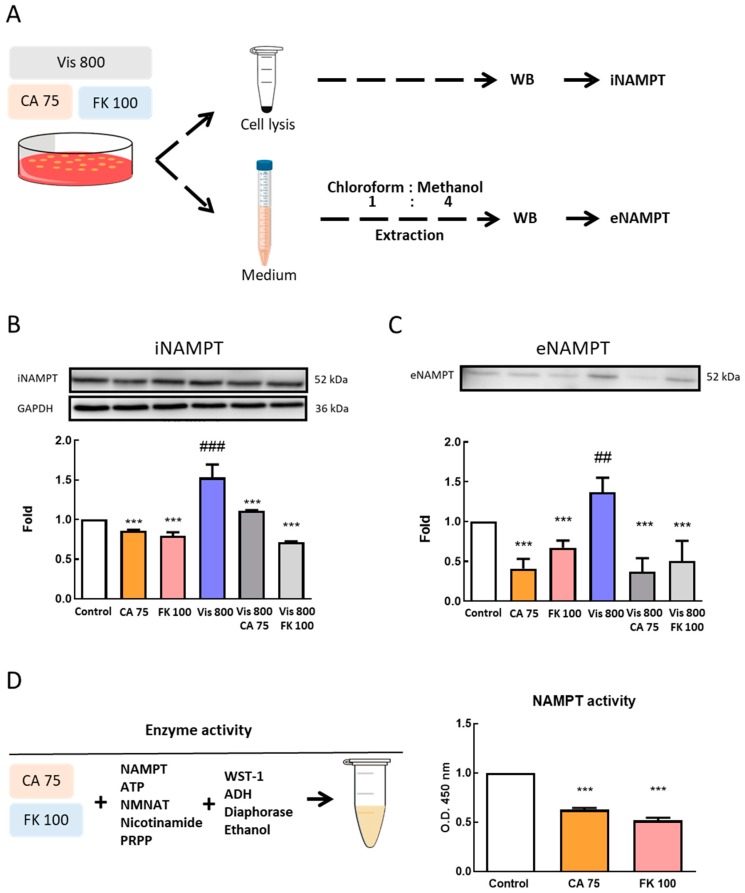
Protein expression of intracellular nicotinamide phosphoribosyl transferase (iNAMPT) and extracellular nicotinamide phosphoribosyl transferase (eNAMPT). (**A**) Sample collection flow chart. (**B**) iNAMPT and (**C**) eNAMPT protein expression. MDA-MB-231-GFP cells were starved for 24 h and then treated with visfatin, cinnamaldehyde (CA), and FK866 for 72 h (*n* = 5), and (**D**) NAMPT activity, the inhibiting effect of CA (75 μM) and FK866 (100 nM) on NAMPT activity. ## *p <* 0.01 compared with the control group. *** *p <* 0.001 compared with the Vis 800 group.

After treatment for 72 h, protein expression of visfatin in the culture medium (extracellular) and cell lysis (intracellular) was analyzed. Cultured medium was collected after 72 h of treatments. Methanol:chloroform (4:1) was used for protein extraction and centrifuged at 14,000 rpm for 5 min. After centrifugation, the pellets were quantified and Western blot was used for further analysis (Figure 5A). The results show that, compared with the control group, the protein expression of intracellular nicotinamide phosphoribosyl transferase iNAMPT (Figure 5B) and extracellular nicotinamide phosphoribosyl transferase eNAMPT (Figure 5C) was significantly increased in the visfatin group (*p* < 0.01). In the combined CA and FK866 group, compared with visfatin, iNAMPT and eNAMPT protein expression was significantly reduced (*p <* 0.001). The activity of visfatin (Figure 5D) was also analyzed using the visfatin activity kit. It was found that both CA and FK866 significantly inhibited the activity of visfatin (*p <* 0.001).

### 3.6. Effect of CA on Visfatin-Induced Proliferation Xenograft Animal Model

#### 3.6.1. Xenograft Animal Model Tumor Change

**Figure 6:**
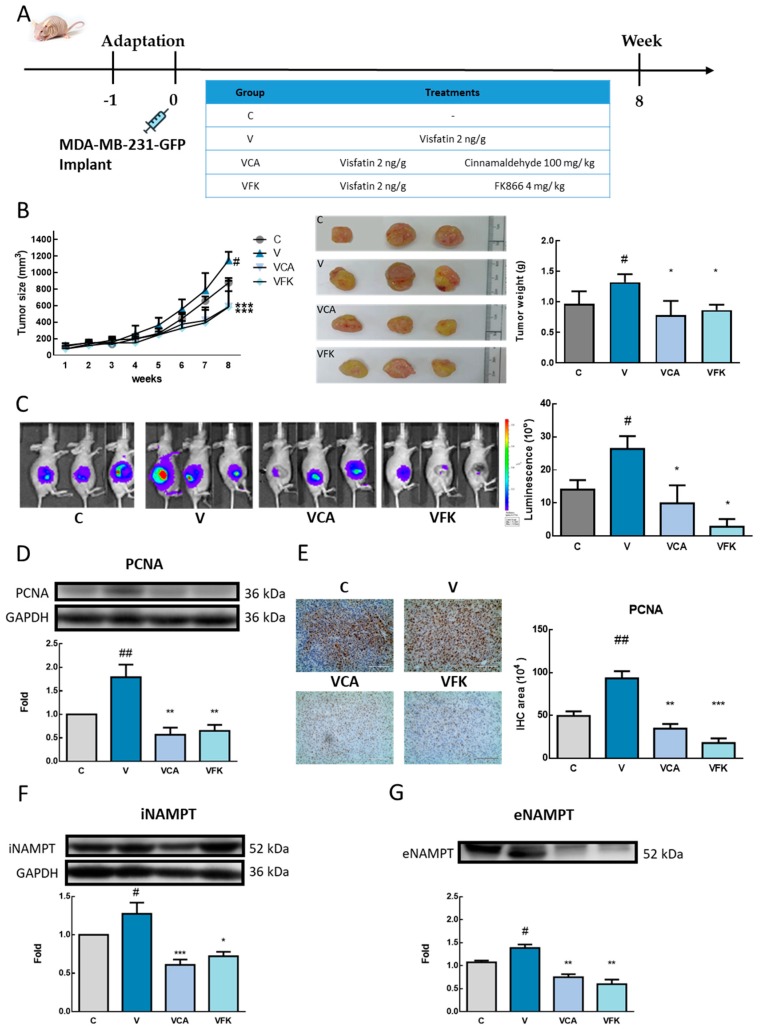
Effects of visfatin, cinnamaldehyde (CA), and FK866 on breast cancer cell proliferation in a xenograft animal model. (**A**) The flow chart and treatment of the xenograft animal model. (**B**) The change of the tumor size, tumor morphology, and weight. (**C**) Total luminescence flux on day 56 after heterotopic tumor cells injection (*n* = 3). (**D**) The change of proliferating cell nuclear antigen (PCNA) protein expression (*n* = 3). (**E**) Immunostaining of the tumor sample. (**F**) intracellular nicotinamide phosphoribosyl transferase (iNAMPT) and (**G**) extracellular nicotinamide phosphoribosyl transferase (eNAMPT) protein expression of the tumor sample (*n* = 3). Control group. V, Visfatin group. VCA, Visfatin + CA group. VFK, Visfatin+ FK866 group. # *p* < 0.05, ## *p* < 0.01 compared with the C group. * *p* < 0.05, ** *p* < 0.01, and *** *p* < 0.001 compared with the V group.

To investigate the effect of CA on inhibiting visfatin-induced tumor proliferation, a xenograft animal model was used. Visfatin (2 ng/g) was administered intraperitoneally (V group) and combined with CA 100 mg/kg (VCA group) or FK866 4 mg/kg (VFK group) (Figure 6A). The animals were sacrificed after the end of the eight-week treatment. Figure S4 shows the body weights and organ weights between the groups. There was no significant difference observed between the groups. In the visfatin group, the size and weight of the tumor after sacrifice were significantly higher than those in the control group, and the combined intervention of CA or FK866 showed weights significantly lower than that of the visfatin group (*p <* 0.05) (Figure 6B).

In the tumor luminescence tests, the visfatin group significantly increased the luminescence signal, compared with the control group, and the combined intervention of CA or FK866 significantly reduced the signal induced by visfatin (*p* < 0.05) (Figure 6C).

#### 3.6.2. Xenograft Animal Model Protein Expression

Western blot analysis of tumor and serum proteins was carried out. The results showed that, when compared with the control group, the presence of proliferating cell nuclear antigen (PCNA) in the visfatin group and the C group significantly increased the expression of PCNA (*p* < 0.01) (Figure 6D). A combination with cinnamaldehyde (CA) (VCA (Visfatin + CA group)) or FK866 (VFK (Visfatin + FK866 group) significantly decreased PCNA protein expression (*p* < 0.01). From the immunostaining of tumors, according to an immunohistochemistry analysis, PCNA (marker of cell proliferation) also showed a consistent result with the findings above (Figure 6E).

Analysis of intracellular (Figure 6F) and extracellular visfatin (serum) (Figure 6G) showed that the V group significantly increased protein expression when compared with the control group (*p* < 0.05), while the levels in the CA (VCA) and FK866 (VFK) groups were significantly lower than in the V group (*p* < 0.01).

## 4. Discussion

The therapeutic strategies for patients with breast cancer are traditionally chemotherapy or radiation therapy. Recently, many studies have examined the effects of the natural compound for adjuvant therapy. CA is very stable at room temperature for 24 h and can be subjected to three cycles of freezing–thawing without significant degradation. As a common spice in many cuisines, CA is widely used for flavoring, and consumption in food can reach 180,000 kg per year in North America [33].

In in vitro studies, according to a previous study, CA caused about 20% cell death at 80 μM on normal human mammary epithelial cells MCF10A [34]. Our results also show that CA would cause normal human epithelial cell death, but at such a high dose that CA caused more serious cell death of MDA-MB-231-GFP breast cancer cells. Converting the in vivo study’s dose to humans, this would be equal to 8.1 mg/kg BW per day [35]. For LD50 values in mice, this was 3.4 g/kg [36]. For intraperitoneal injection (IP) injection, this was 2.318 g/kg in mice.

Cancer cells require a unique energy metabolism process for proliferation. Visfatin, as a metabolic enzyme, plays an important role in cancer cell energy metabolism. Visfatin, which is also known as nicotinamide phosphoribosyl transferase (NAMPT), is a rate-limiting enzyme for the mammalian nicotinamide adenine dinucleotide (NAD) salvage synthesis pathway. NAD is an important product of energy metabolism in cells, participating in energy metabolism, mitochondrial functions, and oxidation reactions. There are three pathways [37] for the synthesis of NAD, including the form starting from tryptophan, the remediation synthesis pathway from nicotinamide to nicotinamide mononucleotide (NMN) to NAD, and the use of dietary nicotine in the Preiss–Handler synthetic pathway. Acid and niacin phosphoribosyl transferase (NAPRT) produce nicotinic acid mononucleotide (NAMN), which is then converted to nicotinic acid-adenine dinucleotide (NAAD) by NAMN transferase (NMNAT), where the salvage synthetic pathway is the primary source of energy for tumor cells. Studies have shown that NAMPT is highly expressed in the serum of patients with gastric cancer, pancreatic cancer, colorectal cancer, breast cancer, and other cancers. It is closely related to the course of these diseases. A previous study has shown that NAMPT and NAD energy metabolism play an important role in cancer progression [7].

Visfatin has intracellular and extracellular forms, which have different functions. Intracellular visfatin (intracellular NAMPT, iNAMPT) can be used for the synthesis of NAD, cancer cell metabolism, and DNA repair [38]. Extracellular visfatin (intracellular nicotinamide phosphoribosyl transferase (iNAMPT), extracellular nicotinamide phosphoribosyl transferase (eNAMPT)) can increase tumor cell metastasis and proliferation, and has demonstrated inflammatory, proangiogenic, and insulin-like effects [8]. Studies have shown that colon cancer, breast cancer, gastric cancer, and endometrial cancer patients have significantly higher serum visfatin concentrations than healthy people [7]. In clinical studies, patients with breast cancer have a significantly higher concentration of visfatin [5]. For advanced evidence in meta-analysis, an association between high visfatin concentrations and increased risk of various cancers has been shown [10]. In our meta-analysis results, we found that visfatin significantly increased the TNM stage and reduced the survival rate in breast cancer. The results showed that visfatin is an important factor in breast cancer progression.

In vitro studies have also shown that visfatin plays an important role in cancer cell proliferation. In previous studies, in endometrial cancer cells [32], visfatin (400 ng/mL) stimulated proliferation and inhibited apoptosis in Ishikawa and KLE cells at 72 h. By adding the phosphoinositide 3-kinases (PI3K) inhibitors (LY294002) and mitogen-activated protein kinase kinase (MEK) inhibitor (U0126), the effects of visfatin were suppressed. Therefore, visfatin was found to promote the progression of endometrial cancer through Protein Kinase B(Akt) /phosphoinositide 3-kinases (PI3K) and extracellular-signal-regulated kinase/ mitogen activated protein kinase (ERK/MAPK) signaling. Previous study examined visfatin treated with breast cancer cell lines MDA-MB-231, MCF7, and T47D for 72 h, and found that doses above 100 ng/mL had a significant proliferative effect [5]. Considering the breast cancer cells MDA-MB-231 and MCF7, visfatin is activated by AKT/PI3K and ERK/MAPK pathways to induce proliferation and inhibit apoptosis [12]. In in vitro studies, our results show that visfatin significantly increased cancer cell proliferation, but a combination with CA and FK866 significantly reduced cancer proliferation.

Studies of natural compounds for reducing visfatin expression are few. Previous studies have shown that curcumin significantly reduced visfatin protein expression [39]. Furthermore, resveratrol treated with HepG2 hepatocarcinoma cells significantly reduced nicotinamide phosphoribosyl transferase (NAMPT) activity [40]. However, there has been no study that has analyzed both the intracellular and extracellular activity of NAMPT. We propose that this paper is the first to observe a natural compound that has an ability to reduce intracellular and extracellular nicotinamide phosphoribosyl transferase (NAMPT) activities.

In vivo studies were conducted in a xenograft model, where visfatin (2 ng/g) was administered intraperitoneally (IP) for four weeks, which promoted Ishikawa xenograft tumor growth displaying a strong proliferation index (Ki-67) [32]. In the breast cancer metastasis xenograft model, the MDA-MB-231 cell line was injected via the tail vein. Interventional visfatin (0.5 mg/kg) by intraperitoneal injection over eight weeks significantly increased tumor size and luminescence intensity [5]. In our study, we used visfatin (2 ng/g) with CA and FK866, administered for eight weeks—the first time a combination of visfatin with a natural compound was used in a xenograft animal model—which significantly reduced proliferation-associated protein expression. Additionally, to clarify the function of extracellular NAMPT in cancer cell proliferation, we conducted an immunohistochemical analysis of PCNA and intracellular and extracellular NAMPT, which showed consistent results. This means that CA and FK866 may reduce extracellular visfatin and, consequently, reduce visfatin-induced cancer cell proliferation. The mechanisms of visfatin-associated and proliferation-associated regulation need further research for confirmation.

## 5. Conclusions

**Figure 7:**
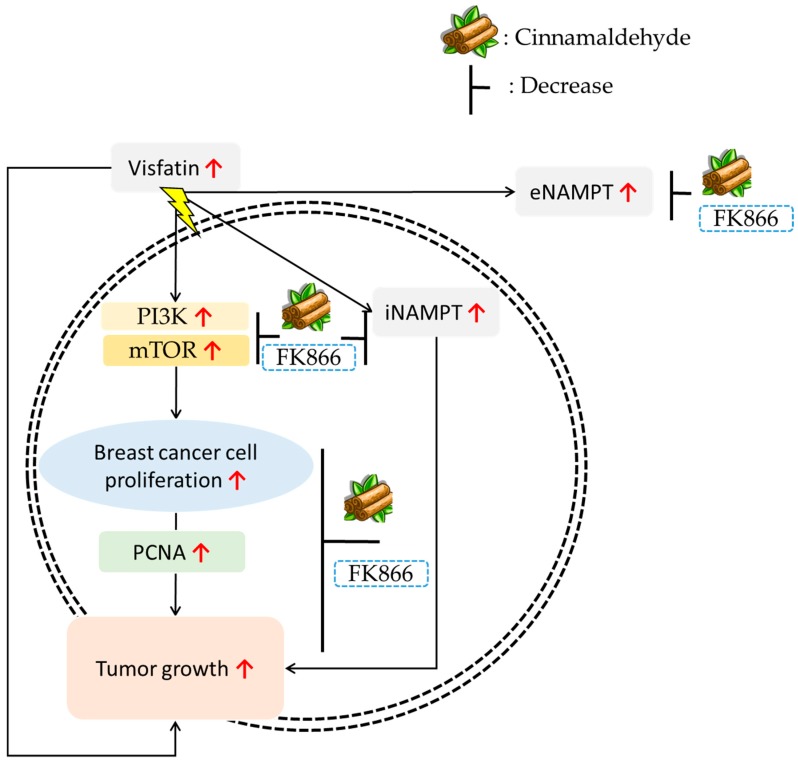
Possible model for the inhibition of tumor growth by cinnamaldehyde and FK866 in visfatin-treated breast cancer cells. In vivo and in vitro study showed that CA inhibited visfatin-induced proliferation, colony formation, and tumor growth by reducing p-mTOR, p-PI3K, PCNA-related protein expression to have an anticancer effect.

In conclusion, our findings indicate that visfatin plays an important role in breast cancer growth and proliferation. Visfatin can enhance iNAMPT, eNAMPT, PI3K, and mTOR protein expression and increase breast cancer cell proliferation. However, intervention by CA or FK866 (visfatin inhibitor) could inhibit (1) visfatin-induced iNAMPT and eNAMPT protein expression, (2) NAMPT enzyme activity, (3) breast cancer cell proliferation, (4) cell proliferative-associated protein expression (PI3K, mTOR, and PCNA), and (5) tumor growth in vivo (Figure 7). Moreover, this is the first time a natural compound has been found to inhibit extracellular and intracellular NAMPT. Therefore, supplementation with CA may aid suppression of the growth of breast carcinoma, which has higher NAMPT expression.

## Supplementary Materials

The following are available online at https://www.mdpi.com/2076-3921/8/12/625/s1. Table S1. Primary antibodies used in this study. Figure S1. Effect of CA on breast cancer cell. Figure S2. Effect of CA on normal epithelial cells and breast cancer cells. Figure S3. Effects of visfatin combined with CA and FK866 on MCF-7. Figure S4. The body weights between the groups. There was no significant difference observed between the groups.
