# Supplementary material for: Dietary Antioxidant Trans-Cinnamaldehyde Reduced Visfatin-Induced Breast Cancer Progression: In Vivo and In Vitro Study
Source: Antioxidants (Basel). 2019 Dec 6;8(12):625. doi: 10.3390/antiox8120625 (PMC6943554; doi:10.3390/antiox8120625)
Supplement: Supplementary file 1 [file antioxidants-08-00625-s001.pdf]

Table S1. Primary antibodies used in this study

Antibody	Brand	Catalog	Dilution
GAPDH	Proteintech	HRP-60004	1:10000
NAMPT	Proteintech	11776-1-AP	1:1000
p-mTOR	Cell signaling	2971S	1:1000
mTOR	Cell signaling	2972S	1:1000
p-PI3K	Cell signaling	4228S	1:1000
PI3K	Cell signaling	4257S	1:1000
PCNA	Cell signaling	13110S	1:1000

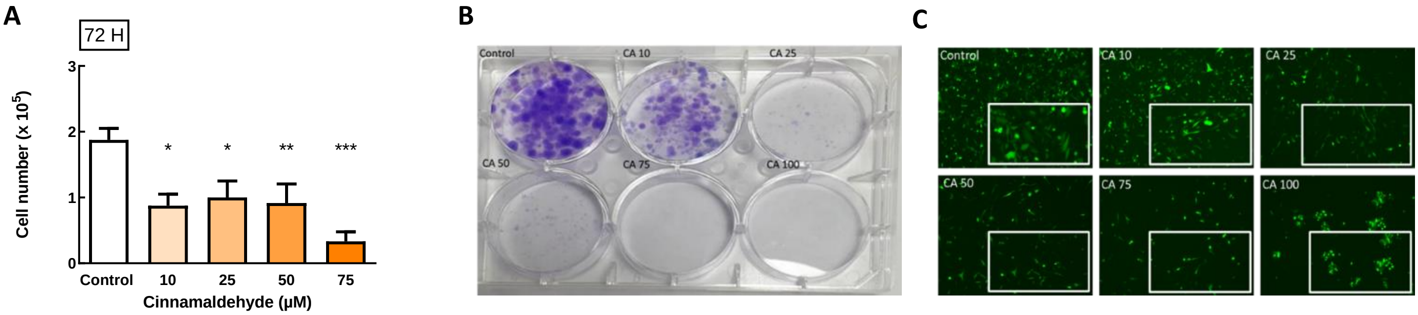

Figure S1. Effect of CA on breast cancer cell (A) Cell counting, treated with CA 72 h; (B) Colony formation, effects of CA on MDA-MB-231-GFP cells after 72 h and maintained for 1 week (C) Cell morphology, treated with CA 72 h. *p < 0.05, **p < 0.01, ***, p < 0.001 compared with control group.

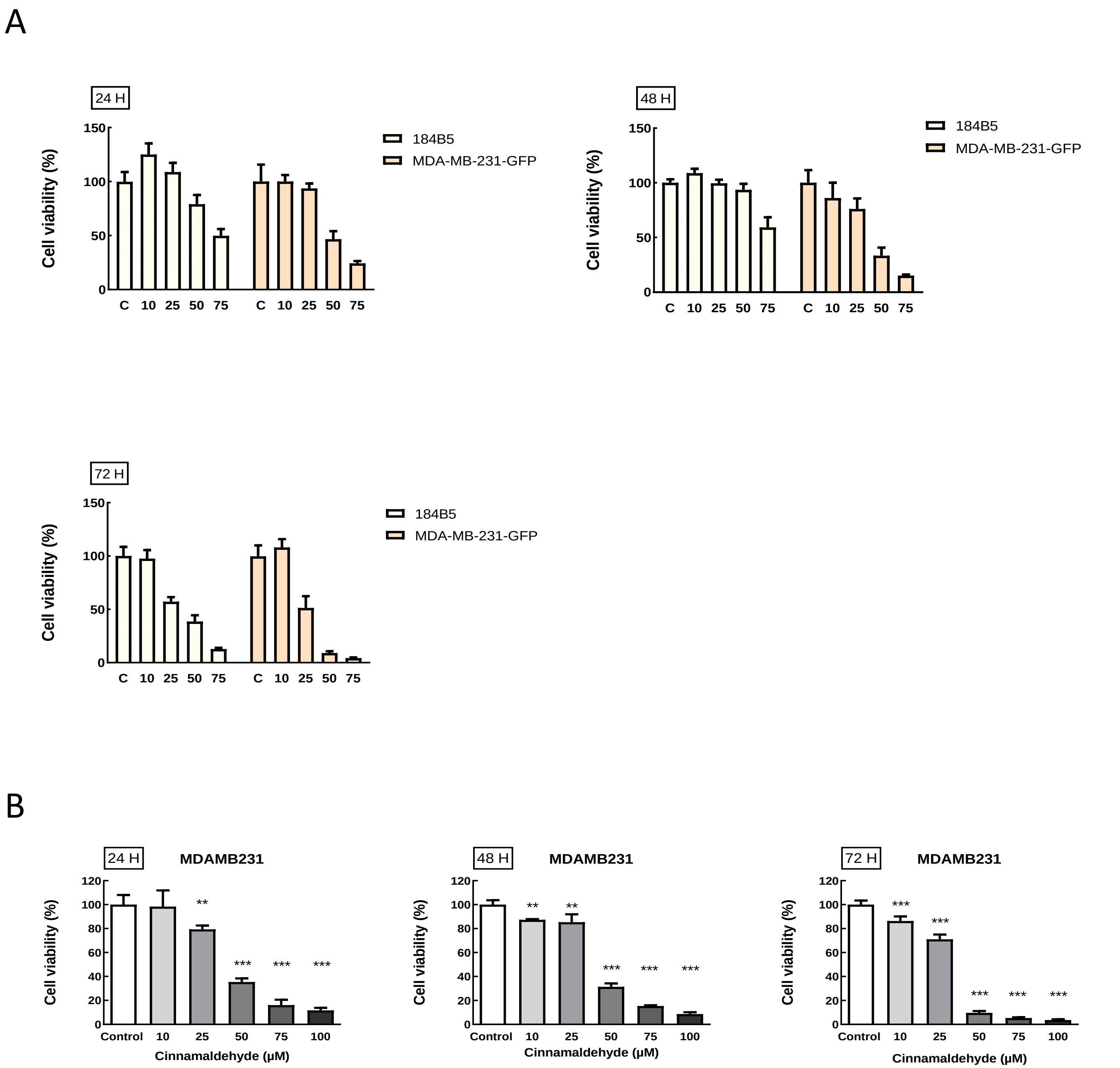

Figure S2. Effect of CA on normal epithelial cell, MDA-MB-231 and MDA-MB-231-GFP breast cancer cell (A) Effects of CA on 184B5 and MDA-MB-231-GFP cells. (B) Effect of CA on MDAMB231 cells. p < 0.05, **p < 0.01, ***, p < 0.001 compared with control group.

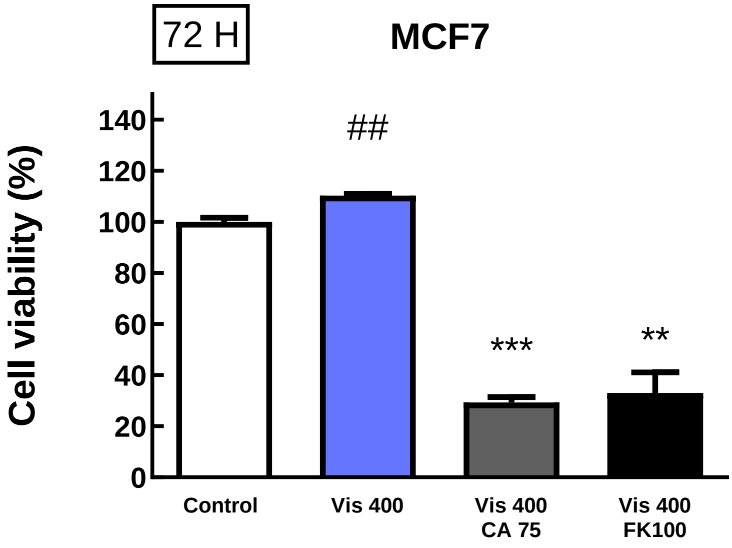

Figure S3. Effects of visfatin combined with CA and FK866 on MCF-7.
##p < 0.01 compared with control group; **p < 0.01, ***, p < 0.001 compared with visfatin group

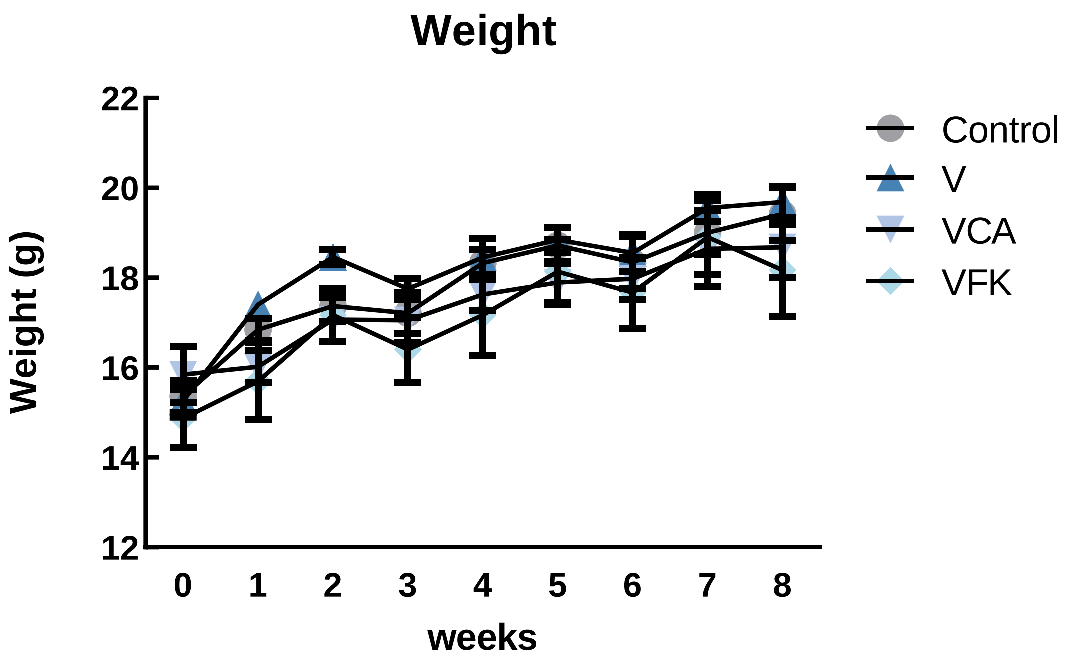

Figure S4. The body weights and organ weights between the groups; there was no significant difference observed between the groups.
